# Experience of the biggest Med School in Mexico during the COVID-19 pandemic

**DOI:** 10.15694/mep.2020.000101.1

**Published:** 2020-05-18

**Authors:** Maria de los Angeles Fernandez-Altuna, Diego Gutierrez Rayon, Mariela Ramirez Resendiz, Patricia Cruz Mendez, Karla Alejandra Tovar Lopez

**Affiliations:** 1 National Autonomous University of Mexico

**Keywords:** education, adaptation to crisis, school´s pandemic experience, school´s administration change, COVID-19

## Abstract

This article was migrated. The article was marked as recommended.

The coronavirus 19 (COVID-19) pandemic affected virtually all activities worldwide. One of them was education, especially Health Sciences. Globally, many medical schools ceased their face-to-face activities and implemented institutional reorganization actions. According to their characteristics and possibilities, institutions adopted diverse strategies and tools to continue providing their services online during this health crisis. These situations implied huge challenges, especially for certain regions, such as Latin America.

This article exposes a quick overview of the pandemic experience at the biggest Mexican Med School (UNAM Med School): forecasting, reorganization, actions, challenges, and learnings.

Among the most challenging situations experienced were: effective communication strategies; resistance to migrate from face-to-face activities to distance activities; technological development; students and teachers training to implement work and study in virtual spaces; students digital gap; internet and computers access; construction and application of online evaluations; online evaluation of practical skills, and the impossibility of maintaining students in clinical clerkships given the pandemic hazardousness.

UNAM Med School was not only reorganized to provide integral care to its community, but it also participated in tasks for the health benefit of Mexico as well as for other countries. We had a huge amount of work, reorganization efforts and creativity resulting in very helpful innovations and new projects.

This health crisis showed the best of our community. Actions will remain along the pandemic period and a progressive reincorporation to in-place activities at the end of the health crisis. Some strategies such as virtual activities within teaching, learning, work, evaluation and research will be maintained.

When this situation ends, we will hopefully have learned and applied that new experiences to improve our Med School, transitioning into a stronger, more united, and enriched community after the crisis caused by this pandemic.

## Introduction

Over the years, human beings have adapted to the different changes that come their way, innovating, creating, and developing measures that allow them to safeguard their needs in adverse environments. This transition has favored transformation and human progress, allowing their environment’s stabilization ( Huremovi′c, 2019).

Currently, society has sought progress through scientific and technological development, and each country has been searching for its development according to its possibilities. For Mexico, assimilating these changes represent a challenge, mainly in education, where marginalization, poverty, and illiteracy hinder training and technological adaptability, a disadvantage that has been noticeable since the early 1980s ( Olmedo, 2010), decreasing national’s innovation and technological improvement.

The coronavirus 19 (COVID-19) pandemic harshly affected the world in several branches of human endeavor ( Bruns, Kraguljac and Bruns, 2020): one of them is the educational sector since the global use of technological tools had not been consolidated ( Daniel, 2020); this sector was also affected with schools and universities closure in 166 countries during March 2020 and impacting more than 91.3% of the world’s student population during April ( UNESCO, 2020) hence generating an educational crisis in countries with limited technological development, to avoid between 2% and 4% of deaths with these measures ( Viner *et al.*, 2020).

New strategies implementation was required to limit the pandemic’s educational effects ( Rachul *et al.*, 2020) ( Taylor *et al.*, 2020), so the United Nations Educational, Scientific and Cultural Organization (UNESCO) recommended planning measures to higher studies institutions to avoid compromising students educational right, and highlighting that medical and public health education institutes must contribute to general population’s health education and promote scientific research.

In Mexico, the National Autonomous University of Mexico (UNAM), a public institution with a student population greater than 360 thousand students at the baccalaureate, undergraduate and postgraduate levels, maintained its education compromise by expanding its knowledge and improving its quality, promoting academic, scientific, cultural, and technological development.

To confront the pandemic under the “social distancing” national policies issued by the Mexican Government, UNAM decided to gradually suspend all its face-to-face activities, which required a special reorganization of all the university’s dependencies.

## UNAM’s pandemic response

• **Verified information**

UNAM’s vehement social commitment, altogether with its scientific and technological progress, allowed to reorganize itself for quickly and accurate truthful information dissemination about the disease. University worked through different technological tools such as web pages, social networks, telephone services, and digital documents to avoid fake news and limit social panic and collective hysteria, especially due to Mexico’s ranking as second in the “misinformation epidemic”, a worrying phenomenon within Mexican population ( UNAM Newsletter, 2020).

• **Academic situation**

Different teaching-learning strategies were implemented to maintain academic activities and continue the curricula contents available with more than 16 thousand virtual classrooms, massive open online courses (MOOCs), Coursera platform, “I learn +” courses, Learning Support Units (UAPA), audios and conferences through Zoom, Google Classroom, Edmodo, Moodle, and Meet, among others. Those resources are available in a “Virtual Campus” ( CUAED, 2019) allowing accessible and significant academic development for its community.

It is undeniable that new generations are better adapted to technological advances, incorporating them in their daily activities, and allowing new pedagogical environments creation; while teachers need to adapt to these teaching processes. Consequently, UNAM together with the Educational Innovation Network’s university members developed a website with educational resources to innovate teaching to face the health crisis ( BUAP, 2020).

• **Institutional innovation**

UNAM convoked its teaching and research population to create innovative proposals against COVID-19 for the benefit of the university’s community and the Mexican population. The results include the implementation of different mathematical model projects on the March’s infectious outbreak, such as the development of a biosensor for rapid and massive detection of the virus and a geographic information platform for COVID-19 evolution in Mexico ( CIGA, 2020), keeping the general population informed on the new cases’ origin, through the use of deep learning algorithms and Big Data techniques, allowing to estimate the number of possible infected persons.

A molecular diagnostic service was launched to carry out tests into the university community, referring the population that tested positive to the different established hospital centers in the city.

UNAM manufactured face masks through a homemade 3D printer to face supplies lack in hospitals nationwide, simultaneously, it contributed with respirators prototypes creation, alcohol gel manufacture, among others.

• **Human Rights Respect**

National social distancing measures (isolation and quarantine) limited physical contact and exposure between people, however, they had considerable repercussions concerning violence, as well as a psychological affectation. For this reason, UNAM provided advice to the general population in the event of gender violence or psychological suffering to help vulnerable groups in confinement, at the same time the Coordination of Gender Studies and Research developed a website with information about gender violence ( CIEG, 2020).

• **UNAM in management first line to combat against COVID-19**

UNAM, being part of the General Health Council of Mexico, contributes in decisions of national policies to combat COVID-19 and actively participates in various organizations whose mission is to deal with COVID-19 pandemic and its health, economic, social, cultural and bioethical consequences.

• **Culture**

UNAM made bibliographic collections available to the public through the National Digital Library of Mexico, as well as the creation of an extensive online artistic-cultural program to promote cultural outreach.

## The UNAM Med School facing the pandemic

The UNAM Med School has a profound commitment to its community, as well as to the Mexican population. In the face of this pandemic, it has reinforced its actions, resulting in more focused attention to the different situations derived from COVID-19.

UNAM Med School has five health undergraduate degrees with more than 8,000 students and nearly 18,000 postgraduate students (medical specializations, master’s degrees, and doctorates), as well as multiple continuing education activities. The academic group includes nearly 5,000 academics, 310 full-time researchers, emeritus, or professors working in the different departments, research and teaching units, peripheral units, and other areas belonging to the Med School ( Fajardo, 2019).

In the face of the pandemic, our Med School maintained its commitment to meet the needs of its community and society by implementing and launching several projects to help and deal with the health crisis and its repercussions on education, research and medical care.

Some institutional actions that were implemented as a response from UNAM′s Med School to the pandemic will be described. We will divide them into two parts: those focused on the academic-administrative community of our Med School and those aimed to the general population.

## 1. Some UNAM Med School institutional actions for our community in response to the situation caused by COVID-19

Since the COVID-19 pandemic was declared, UNAM’s Med School installed alcohol-gel dispensers in all its buildings as a sanitation action and published posters with hygiene instructions and disease infographics. It was also implemented an intensive communication campaign on the subject to sensitize and inform the entire community. Gradually, “social distancing” measures were implemented, the sanitation of academic and work areas was reinforced, and started planning how to continue academic, research, artistic-cultural, and administrative tasks at distance.

Finally, on March 17, 2020, it was decided to suspend all face-to-face activities (classes, conferences, academic, cultural and artistic events, etc.). Some of the activities were carried out remotely.

Due to the concern of academic monitoring, the “MediTIC” project emerged. It is a new digital platform created to continue teaching and learning through tutorials, academic tools, and distance educational offerings, providing medical, linguistic, technological, and humanistic knowledge through an interactive and easily accessible platform for the five bachelor’s degrees, aimed at students, teachers, health professionals, and the general public ( MediTic, 2020).

To continue providing academic services ( Sabzwari, 2020), virtual classrooms and academic consultancies were set up to reinforce the contents of the websites of each academic department of the Med School. All exams and evaluations were implemented online, including the professional access exam. A full digital couching program was developed in order to prepare students for the new online exams.

At this stage, the teachers’ and students’ training in this online new educational modality, the unequal access to the internet that characterizes the country, internet connection problems, and preparation and management of a whole battery of *ad hoc* new evaluations to be applied remotely were the main challenges. Another special challenge was evaluating practical skills.

Medical students who were attending hospitals and clinics were sent home to safely continue remote learning activities. Several online workshops were held, including one on the use of personal protection equipment. Based on Moodle, several clinic cases for discussion were created to work with 3th, 4 ^th^ and 5th grade students.

A web page was exclusively developed to provide information on COVID-19 where a large amount of material (videoconferences, infographics, real-time statistics, digital documents, etc.) was included, to facilitate understanding of the disease, hygiene measures, and the pandemic monitoring, among other aspects ( COVID-19, 2020).

Communication with Med School′s community was specially reinforced from de moment we knew about the pandemic, even before it reached our country. Communications were intensified through our different official social networks (Facebook, Twitter, TikTok), as well as through emails, posters, and various applications for cell phones. A new institutional instant messaging service (Telegram) was implemented to communicate important notices to the entire community 24 hours a day on various aspects during the pandemic.

Besides, various press conferences, conferences, and symposia have been held for doctors, as well as for the general population, on several topics related to the pandemic.

To carry out a daily community monitoring of the student, faculty and researcher′s population and their families, a health survey was launched with the registration of risk factors, presentation of symptoms, as well as the preventive and sanitary measures used in the home and work, allowing a breakdown according to sex, age, municipality, number of co-inhabitants per household, enrollment degree and hospital headquarters ( COVID-19, 2020). Since the 4 weeks it has been implemented, more than 54 thousand surveys had been completed. 20% of this population still leaves home for reasons such as food, medicines or going to work, the vast majority (90%) wearing face masks. This survey also has been made possible to monitor people with certain symptoms and send them to seek medical care.

An online consultation service was made available to the community by the Department of Psychiatry and Mental Health ( DPMH, 2020), to help the university’s population in case of relapses, increase or appearance of psychological symptoms caused by this health crisis ( Liang *et al.*, 2020) ( Zhai and Du, 2020).

To support our community in aspects as important as physical health and healthy spare, many online yoga classes, art workshops and cultural activities were created and promoted.

Mathematical and epidemiological models, various research protocols on COVID-19 were developed at hospitals (clinical studies of diagnosis and treatment and basic sciences approaches). Many of these investigations were held in collaboration with other educational centers, foundations and hospitals.

Regarding school administration, all universities and schools must review their processes, requirements, and regulations on school evaluation and progression ( McKimm *et al.*, 2020); consequently, the med school’s administration had to adapt and migrate from face-to-face services to online services for more than 25 thousand students during the health crisis. These adaptations ranged from enrollment, document delivery to various evaluations, and even professional exams and degrees, all of the administrative processes that are relevant in students’ academic trajectories and of the universities themselves.

Another great challenge for the Remote School Academic Administration was the management of confidential information such as personal data and school documentation through the web, as well as the extraction of information from the databases of the faculty servers from the academic-administrative staff’s home (home office).

Despite the challenges, the result of the strategic planning of human and technological resources facilitated the linking of the different areas, allowing an efficient school academic administration of more than 25 thousand undergraduate and postgraduate students.

The Med school’s financial administration continues its operations remotely. In a national and global environment characterized by job losses and financial crisis, all the collaborators of the faculty have continued to receive their salaries regularly.

In the face of the COVID-19 pandemic, an adaptability process was required, with the implementation of completely unknown measures, adding to the challenges of personnel organization, generation gaps, effective communication, and the use of various information and communication technologies. Also, special psychological “people-focused” organizational techniques were required, given the vulnerability of individuals to the uncertainty and dangers of a pandemic.

Work from home (WFH) or home office turns into a great organizational method that brings benefits, however, few countries in Latin America use it regularly ( Aquije, 2018). It is known that WFH usually involves a substantial increase in the amount of work and that people tend to resist change.

Initially, for our Med School it was difficult getting used to the change, but in a few weeks, both virtual meeting platforms (and other technological challenges), schedule organization and new processes for home office and digital learning were mastered.

During the health crisis, the WFH and many digital and online strategies allowed Med School community to continue with the academic, research, cultural and effective school administration activities.

In addition to keep providing services to the community, this entire new adaptation process provided an extra benefit that was not foreseen: consolidation of a new network of psychological and social support for the work teams and the whole Med School community.

## 2. Some UNAM Med School actions aimed to the general population and other countries in response to the situation caused by COVID-19

To provide security, protection, and care to the Mexican population, UNAM Med School implemented a “call center” and a “chat center” to answer questions and provide medical guidance to the general population.

To join the efforts of the Mexican government given the need to serve the population affected by COVID-19, our Med School actively participated in the creation of the Temporary Hospital Unit (840 beds) in the facilities of the Citi-Banamex Congress Center in Mexico City, in collaboration with many Altruistic Foundations ( Hernández, 2020).

Given the shortage of personal protective equipment in hospitals, a donation campaign was launched with the UNAM Foundation to obtain protection kits for doctors in training at the postgraduate level who are in hospital centers, providing them with more than 400 000 equipment kits to attend to the population infected with COVID-19 ( Chirino, 2020).

The Med School, through its Traveler Preventive Care Clinic and its Research Laboratory for Infectious Diseases, made the Diagnostic Center for COVID-19 available to the community at Mexico City International Airport, which provides diagnosis and monitoring ( Diagnostic Center COVID-19, 2020).

In collaboration with the BBVA Mexico Foundation and the Technological de Monterrey School of Medicine and Health Sciences, different digital educational programs were developed, including endotracheal intubation, basic training in airway management, as well as medical skills for the pandemic, programs that allow proper training to provide quality care.

To share experiences with other countries in the region, UNAM’s Med School actively participated in a series of webinars organized by the Union of Universities of Latin America and the Caribbean (UDUAL): “The role of medical schools in the face of the COVID-19 contingency”, “Challenges of Latin American Universities in the context of the COVID-19 pandemic”, among others ( UDUAL, 2017).

To assess the national situation concerning the inequality of economic resources and food shortages, the university worked with the home food delivery application “Rappi” and other allies assessing the nutritional part of the altruistic initiative “a sack” to provide food for vulnerable families.

## “The come back”

It is still uncertain when will be the right moment to “come back” to face-to-face activities for our Med School and what is gonna be “the new normal”.

The impact and recovery plan to be followed by other universities equally seeking strategies for the benefit of their students is unknown ( Ries & Wagner, 2020) and work continues for the mental health needs after the major consequences during and at the end of the pandemic ( Pfefferbaum & North, 2020).

We are changing our academic calendar according to modifications on pandemic′s situation in our country. Mexico is now beginning pandemic′s phase 3, we still have a lot to endure, particularly on health, mental and economic issues.

We have been acting all this time on behalf our students, faculty, administrative personnel and our country. There is still a long way to go. We will continue to evaluate day to day and act accordingly, always looking the best for our students and faculty.

It seems clear that our Med School will have to take care of many aspects of its community because of the pandemic′s damage: academic issues, mental health, survivor′s condition and mourning for our losts, post-traumatic syndrome, national and global economic problems affecting our country, fear to new waves of the disease, and so on.

After this ferocious pandemic, we will have to take special care of the scars and to embrace the new community we will be.

## Conclusion

The health crisis carried a high degree of uncertainty and implies the need for adaptations and innovations that are not easily achieved.

In the case of UNAM′s Med School, the response was swift and forceful, based on the protection of its community, and aimed at continuing with its substantive functions.

The reorganization of goals and processes supported by technological tools allowed to continue with teaching, research, culture and leisure activities, although it was in a different way.

Personal relationships between members of the community were also substantially enriched, highlighting the altruistic vocation and the deep sense of solidarity and service in response to a tragic health success.

The organization and adaptability of UNAM′s Med School allowed the implementation of multiple strategies facilitating resolution of the educational and administrative challenges caused by the pandemic, as well as the creation of new projects for the protection of their academic community and the general Mexican population.

Providing concrete and truthful information about the pandemic to different population groups allowed giving a certain degree of certainty by providing general knowledge and solving their doubts.

We still have to improve, but we have learned that effective communication and respect are crucial to keep moving forward.

It is known that the consequences of this pandemic (political, economic, cultural, personal, bioethical and social) will be enormous, however, it requires innovations and the joining of efforts to overcome the problems that are coming.

Mexico is currently beginning phase 3 of the pandemic so we continue to prepare for the difficult days to come. UNAM’s Med School is ready to continue advancing and reinventing itself during this pandemic to fulfill its mission and vision.

The staggered return to face-to-face work should be planned carefully from this moment and be fully operative when the health crisis ends.

We believe that when this situation ends, we will apply this new lessons and experiences to improve our Med School, transitioning into a stronger, united, and enriched community in the face of the crisis caused by a pandemic.

## Take Home Messages

• Med Schools have to adapt to provide educational services during a global health crisis.
• In our school the most challenging situations were&nbsp;effective communication, resistance to migrate from face-to-face activities to distance activities; technological development; training to implement work and study in virtual spaces; digital gap, computer and internet access; online evaluations; practical skills online evaluation and students withdrawal from clinical clerkships.
• The use of technological tools facilitates the task, but it must be accompanied by effective communication.
• What the educational institution learned during the pandemic must be used to improve and enrich its community in all the possible approaches.

## Notes On Contributors

**María de los Angeles Fernandez-Altuna**. General Physician and PhD in Sociomedical Sciences. Currently Public Health and Basic and clinical Sciences Integration teacher. Vice Dean for Academic Management Affairs at National Autonomous University of Mexico’s Med School. ORCID https://orcid.org/0000-0002-7990-3856

**Diego Gutierrez Rayon**. General Physician, head of Integration, Information and Data Analysis Unit at the National Autonomous University of Mexico’s Med School Academic Management Affairs Secretariat. ORCID https://orcid.org/0000-0003-1063-7075

**Mariela Ramirez Resendiz**. General Physician. Head of the Undergraduate Unit at the National Autonomous University of Mexico’s Med School Academic Management Affairs Secretariat. ORCID https://orcid.org/0000-0003-2418-6455

**Patricia Cruz Mendez**. General Physician. Currently works at the Re-engineering and Sustantive Processes Improvement Area at the National Autonomous University of Mexico’s Med School Academic Management Affairs Secretariat. ORCID https://orcid.org/0000-0001-6024-4136

**Karla Alejandra Tovar Lopez**. General Physician. Currently contributes to the Re-engineering and Sustantive Processes Improvement Area at the National Autonomous University of Mexico’s Med School Academic Management Affairs Secretariat. ORCID https://orcid.org/0000-0002-2212-7884
