# Experience of the biggest School of Medicine in Mexico during the COVID-19 pandemic

**DOI:** 10.15694/mep.2020.000101.2

**Published:** 2020-11-06

**Authors:** Maria de los Angeles Fernandez-Altuna, Diego Gutierrez Rayon, Mariela Ramirez Resendiz, Patricia Cruz Mendez, Karla Alejandra Tovar Lopez

**Affiliations:** 1 National Autonomous University of Mexico

**Keywords:** education, adaptation to crisis, school´s pandemic experience, school´s administration change, COVID-19

## Abstract

This article was migrated. The article was marked as recommended.

The coronavirus 19 disease (COVID-19) pandemic affected virtually all activities worldwide. One of them was education, especially Health Sciences. In the world, many medical schools ceased their face-to-face activities and implemented institutional reorganization actions. According to their characteristics and possibilities, institutions adopted different strategies and tools to continue providing their services online during this health crisis. These situations implied enormous challenges, especially for specific regions, such as Latin America.

This article exposes a quick overview of the pandemic experience at the biggest Mexican School of Medicine (UNAM School of Medicine): forecasting, reorganization, actions, challenges, and learnings.

Among the most challenging situations experienced were: effective communication strategies; resistance to migrating from face-to-face activities to remote activities; technological development; students and teachers training to implement work and study in virtual spaces; students digital gap; internet and computers access; construction and application of online evaluations; online evaluation of practical skills, and the impossibility of maintaining students in clinical clerkships given the pandemic risks.

UNAM School of Medicine reorganized to provide integral care to its community, but it also participated in tasks for Mexico’s health and other countries’ health benefits. We had a great amount of work, reorganization efforts, and creativity resulting in efficient innovations and new projects.

This health crisis showed the best in our community. Actions will remain along the pandemic period and a progressive reincorporation to in-place activities at the end of the health crisis. Some strategies, such as remote activities within teaching, learning, work, evaluation, and research, will be maintained.

When this situation ends, we will hopefully have learned and applied those new experiences to improve our School of Medicine, transitioning into a more robust, more united, and enriched community after the crisis caused by this pandemic.

## Introduction

Over the years, human beings have adapted to the different changes that come their way, innovating, creating, and developing measures to safeguard their needs in adverse environments. This transition has favored transformation and human progress, allowing their environment’s stabilization (Huremovi’c, 2019).

Currently, society has sought progress through scientific and technological development. Each country has been searching for its development according to its possibilities. For Mexico, assimilating these changes represent a challenge, mainly in education, where marginalization, poverty, and illiteracy hinder training and technological adaptability, a disadvantage that has been noticeable since the early 1980s ( Olmedo, 2010), decreasing national innovation and technological improvement.

The coronavirus 19 disease (COVID-19) pandemic harshly affected the world in several branches of human endeavor ( Bruns, Kraguljac and Bruns, 2020): one of them is the educational sector with the global lack of consolidation of technological tools ( Daniel, 2020). This sector was also affected with schools and universities closure in 166 countries during March 2020 and impacting more than 91.3% of the world’s student population during April (UNESCO, 2020) hence generating an educational crisis in countries with limited technological development, to avoid between 2% and 4% of deaths with these measures ( Viner *et al*., 2020).

New strategies were required to limit the pandemic’s educational effects ( Rachul *et al*., 2020) ( Taylor *et al*., 2020), so UNESCO recommended planning measures to higher studies institutions to avoid compromising students educational right; highlighting that medical and public health education institutes must contribute to general population’s health education and promote scientific research.

In Mexico, the National Autonomous University of Mexico (UNAM), a public institution with a student population greater than 360 thousand students at the baccalaureate, undergraduate, and postgraduate levels, maintained its education compromise by expanding its knowledge and improving its quality, promoting academic, scientific, cultural, and technological development.

UNAM, as a social agent facing globalization and emerging and environmental challenges, seeks to promote social accountability through specific and efficient responses to crises, being this a vital tool to respond to changes ( El-Kassar, Makki, and Gonzalez, 2019). Currently, the interest of any individual or organization to assume responsibility for the effects of their actions in the community is known as social accountability ( Gauca and Dragan, 2017). To highlight that it allows integrating and balancing university functions, transforming them into fields of action, promoting social cohesion in challenging times ( Buenaño, Maldonado and Benavides, 2018).

To confront the pandemic under the “social distancing” national policies issued by the Mexican Government, UNAM decided to gradually suspend all its face-to-face activities, which required a specific reorganization of all the university’s dependencies.

## UNAM’s pandemic response

• **Verified Information**

UNAM’s fierce social commitment, altogether with its scientific and technological progress, allowed it to reorganize itself for quick and accurate, truthful information dissemination about the disease. University worked through different technological tools such as web pages, social networks, telephone services, and digital documents to avoid fake news and limit social panic and collective hysteria, primarily due to Mexico’s ranking as second in the “misinformation epidemic”, a worrying phenomenon within Mexican population (UNAM Newsletter, 2020).

• **Academic Situation**

UNAM implemented different teaching-learning strategies to maintain academic activities and continue the curricula contents available with more than 16 thousand virtual classrooms, massive open online courses (MOOCs), Coursera platform, “I learn +” courses, Learning Support Units (UAPA), audios and conferences through Zoom, Google Classroom, Edmodo, Moodle, and Meet, among others. Those resources are available in a “Virtual Campus” ( CUAED, 2019), allowing accessible and significant academic development for its community.

It is undeniable that new generations are better adapted to technological advances, incorporating them in their daily activities, and allowing new pedagogical environments creation; while teachers need to adapt to these teaching processes. Consequently, UNAM, together with the Educational Innovation Network’s university members, developed a website with educational resources to innovate teaching to face the health crisis ( BUAP, 2020).

• **Institutional Innovation**

UNAM convoked its teaching and research population to create innovative proposals against COVID-19 for the benefit of the university’s community and the Mexican population. The results include the implementation of different mathematical model projects on the March’s infectious outbreak, such as the development of a biosensor for rapid and massive detection of the virus and a geographic information platform for COVID-19’s evolution in Mexico ( CIGA, 2020), keeping the general population informed on the new cases’ origin, through the use of deep learning algorithms and Big Data techniques, allowing to estimate the number of possible infected persons.

The institution also launched a molecular diagnostic service to carry out tests into the university community, referring to the population that tested positive to the different established hospital centers in the city.

UNAM manufactured face masks through a homemade 3D printer to face supply shortages in hospitals nationwide; simultaneously, it contributed with respirators prototypes creation, alcohol gel manufacture, among others.

• **Human Rights Respect**

National social distancing measures (isolation and quarantine) limited physical contact and exposure between people; however, they had considerable repercussions concerning violence, as well as a psychological affectation. For this reason, UNAM provided advice to the general population in the event of gender violence or psychological suffering to help vulnerable groups in confinement; at the same time, the Coordination of Gender Studies and Research developed a website with information about gender violence ( CIEG, 2020).

• **UNAM in Management First Line to Combat against COVID-19**

UNAM, being part of the General Health Council of Mexico, contributes in decisions of national policies to combat COVID-19 and actively participates in various organizations whose mission is to deal with COVID-19 pandemic and its health, economic, social, cultural and bioethical consequences.

• **Culture**

UNAM made bibliographic collections available to the public through the National Digital Library of Mexico and created an extensive online artistic-cultural program to promote cultural outreach.

## The UNAM School of Medicine facing the pandemic

The UNAM School of Medicine has a profound commitment to its community and the Mexican population. In the face of this pandemic, it has reinforced its actions, resulting in more focused attention to the different situations derived from COVID-19.

UNAM School of Medicine has five undergraduate health degrees, with more than 8,000 students and nearly 18,000 postgraduate students (medical specializations, master’s degrees, and doctorates), as well as multiple continuing education activities. The academic group includes nearly 5,000 academics, 310 full-time researchers, emeritus, or professors working in the different departments, research and teaching units, peripheral units, and other areas belonging to the School of Medicine ( Fajardo, 2019).

In the face of the pandemic, our School of Medicine maintained its commitment to meet the needs of its community and society by implementing and launching several projects to help and deal with the health crisis and its repercussions on education, research, and medical care.

Since the COVID-19 pandemic was declared, UNAM School of Medicine implemented “social distancing” measures, reinforced the sanitation of academic and work areas, and started planning how to continue academic, research, artistic-cultural, and administrative tasks remotely. Finally, on March 17 ^th^. 2020, it was decided to suspend all face-to-face activities (classes, conferences, academic, cultural, and artistic events, among others.). Some of the activities transformed into remote resources ( Sabzwari, 2020).

This section will mention some institutional actions implemented as a response from UNAM School of Medicine to the pandemic. We will divide them into two parts: those focused on the academic-administrative community of our School and those for the general population. Table 1 summarizes some of those actions.

**Table 1.** Institutional actions of the UNAM School of Medicine in response to the COVID-19 pandemic.

Actions focused on the UNAM School of Medicine Community	
**Academic**	• A full digital coaching and learning program to prepare students and teachers for the new online activities and exams. • Virtual classrooms and academic consultancies were set up to reinforce the contents of each website of the academic departments. • Medical students who were attending hospitals and clinics were sent home. Remote learning by online workshops for students of the third, fourth and fifth grade during their confinement at home. All exams and evaluations were implemented online. • Creation of the “MediTIC” digital platform.
**Communication**	• A web page exclusively developed to provide information on COVID-19. • Reinforced communication through official social networks (Facebook, Twitter, TikTok), and a new institutional instant messaging service (Telegram) to communicate important news. • Community monitoring through a health survey. • Periodic online publications on COVID-19 topics (newsletters, magazines, etc).
**General wellness**	• Online yoga, zumba, music and mediation classes, art workshops, and many other cultural activities to support our community. • Special online activities to promote mental health.
**Administrative**	• Provide online academic-administrative services to its educational population (25,000 undergraduate and graduate students). • Adaptation for the remote extraction of databases from the School computer servers. • Financial administration operations were made remotely. • Implementation of “work at home”: effective and organized.
**Actions focused on the general population**	
**Medical care**	• “Call and chat center” to answer questions and provide medical guidance on COVID-19 for the general population. • Diagnostic Center for COVID-19 at Mexico City International Airport. • Participation in the creation of the Temporary Hospital Unit (840 beds) in the Citi-Banamex Congress Center facilities in Mexico City, in collaboration with many Altruistic Foundations. • Development of mathematical and epidemiological models and several clinical research protocols on COVID-19.
**Teaching and Outreach**	• Webinars for physicians and general population on COVID-19 topics. • Press conferences. • Digital educational programs in collaboration with the BBVA Mexico Foundation and the Technological of Monterrey School of Medicine and Health Sciences. • Participation in webinars organized by the Union of Universities of Latin America and the Caribbean (UDUAL) and other universities.
**Altruistic**	• A donation campaign (with the UNAM Foundation) to obtain protection kits for doctors in training at the postgraduate level in hospital centers with COVID-19 patients. • Assessment to the home food delivery application “Rappi” and other allies for nutritional aspects for the philanthropic initiative “a sack” to provide food for vulnerable families.

## I. Some UNAM School of Medicine institutional actions for its community in response to the situation caused by COVID-19

To continue providing academic services, virtual classrooms and academic consultancies were set up to reinforce the contents of the websites of each academic department of our School. All exams and evaluations were implemented online. A full digital couching program was developed in order to prepare students for the new online exams.

At this stage, the teachers’ and students’ training in this online new educational modality, the unequal access to the internet that characterizes the country, internet connection problems, and preparation and management of a whole battery of *ad hoc* new evaluations to be applied remotely were the main challenges. Another special challenge was evaluating practical skills.

Due to the concern of academic monitoring an innovative academic implementation emerged: the “MediTIC” project. It is a new digital platform specially created to continue teaching and learning through tutorials, academic tools, and distance educational offerings, providing medical, linguistic, technological, and humanistic knowledge through an interactive and easily accessible platform for the five bachelor’s degrees, aimed at students, teachers, health professionals, and the general public ( MediTIC, 2020).

Medical students who were attending hospitals and clinics were sent home to safely continue remote learning activities. Several online workshops were held, including one on the use of personal protection equipment. Based on Moodle, several clinic cases for discussion were created to work with 3th, 4 ^th^ and 5th grade students.

A web page was exclusively developed to provide information on COVID-19 where a large amount of material (videoconferences, infographics, real-time statistics, and digital documents, among others.) was included, to facilitate understanding of the disease, hygiene measures, and the pandemic monitoring, among other aspects ( COVID-19, 2020).

The UNAM School of Medicine performed community monitoring of the student, faculty, and researcher’s population and their families through a health survey. This survey registered risk factors, symptoms, and the preventive and sanitary measures used in the home and work, allowing a breakdown according to sex, age, municipality, number of co-inhabitants per household, enrollment degree, and hospital headquarters ( COVID-19, 2020). In its first four weeks, it obtained more than 54 thousand responses. 20% of this population still leaves confinement for reasons such as food and medicines obtention, or work; the vast majority (90%) wear face masks. This survey also has been made possible to monitor people with specific symptoms and send them to seek medical care. Recently, a mental health section was incorporated.

Communication with School of Medicine community was specially reinforced from de moment we knew about the pandemic, even before it reached our country. Communications were intensified through our different official social networks (Facebook, Twitter, TikTok), as well as through emails, posters, and several applications for cell phones. A new institutional instant messaging service (Telegram) was implemented to communicate important information to the community during the pandemic.

In order to inform other health professionals as well as for the general population, several press conferences, symposia, newsletters, and other online activities were held to discuss or inform on several topics related to the pandemic.

This entire new adaptation process provided an extra benefit not foreseen, consolidation of a new network of psychological and social support for the work teams and the university community through an online consultation service that was made available to the community by the Department of Psychiatry and Mental Health ( DPMH, 2020) before the mental problems observed in this type of crisis ( Liang *et al*., 2020) ( Zhai and Du, 2020).

Mathematical and epidemiological models, several research protocols on COVID-19 were developed at hospitals (clinical studies of diagnosis and treatment, and basic science approaches). Many of these investigations were held in collaboration with other educational centers, foundations and hospitals.

Regarding school administration, all universities and schools must review their processes, requirements, and regulations on evaluation and progression ( McKimm *et al*., 2020); consequently, the School of Medicine administration had to adapt and migrate from face-to-face services to online services for more than 25 thousand students during the health crisis. These adaptations ranged from enrollment, document delivery to various evaluations, and even professional exams and degrees. All these administrative processes are relevant for students’ academic trajectories and for the universities themselves.

Our School’s financial administration continued its operations remotely. In a national and global environment characterized by job losses and financial crisis, all the collaborators of the School have continued to receive their salaries regularly.

In the face of the COVID-19 pandemic, an adaptability process was required with the implementation of entirely unknown measures, adding to the challenges of personnel organization ( Aquije, 2018), generation gaps, effective communication, and the use of several information and communication technologies. Also, special psychological “people-focused” organizational techniques were required, given the vulnerability of individuals to the uncertainty and dangers of a pandemic.

Initially, for our School of Medicine it was difficult getting used to the change, but the community tried hard to adapt to virtual meeting platforms (and other technological challenges), schedule organization, and new processes for home office and digital learning. However, the implementations carried out by the School of Medicine allowed continuing with the academic, research, cultural, and effective school administration activities.

## II. Some UNAM School of Medicine actions for the general population in response to the situation caused by COVID-19

To provide security, protection, and care to the Mexican population, UNAM School of Medicine implemented a “call center” and a “chat center” to answer questions and provide medical guidance to the general population.

To join the efforts of the Mexican government given the need to serve the population affected by COVID-19, our School actively participated in the creation of the Temporary Hospital Unit (840 beds) in the facilities of the Citi-Banamex Congress Center in Mexico City, in collaboration with many Altruistic Foundations.

Given the shortage of personal protective equipment in hospitals, a donation campaign was launched with the UNAM Foundation to obtain protection kits for doctors in training at the postgraduate level who are in hospital centers, providing them with more than 500 000 equipment kits.

The UNAM School of Medicine, through its Traveler Preventive Care Clinic and its Research Laboratory for Infectious Diseases, made the Diagnostic Center for COVID-19 available to the community at Mexico City International Airport, which provides diagnosis and monitoring.

In collaboration with the BBVA Mexico Foundation and the Tecnologico de Monterrey School of Medicine and Health Sciences, different digital educational programs were developed, including endotracheal intubation, basic training in airway management, as well as other medical skills for the pandemic, programs that allow better training to provide quality care.

To share experiences with other countries in the region, UNAM School of Medicine actively participated in a series of webinars organized by the Union of Universities of Latin America and the Caribbean (UDUAL): “The role of medical schools in the face of the COVID-19 contingency”, “Challenges of Latin American Universities in the context of the COVID-19 pandemic”, among others.

To assess the national situation concerning the inequality of economic resources and food, our School worked with the home food delivery application “Rappi” and other allies assessing the nutritional part of the altruistic initiative “A sack” to provide food for vulnerable families.

## “The comeback”

It is still uncertain when will be the right moment to “come back” to face-to-face activities for our School of Medicine and what will be “the new normal”.

The impact and recovery plan to be followed by other universities equally seeking strategies for the benefit of their students are unknown ( Ries and Wagner, 2020) and work continues for the mental health needs after the significant consequences during and at the end of the pandemic ( Pfefferbaum and North, 2020).

We are changing our academic calendar according to modifications to the pandemic situation in our country. Mexico is now beginning pandemic’s phase 3, we still have a lot to endure, particularly on health, mental and economic issues.

We have been acting all this time on behalf of our students, faculty, administrative personnel, and country. There is still a long way to go. We will continue to evaluate day to day and act accordingly, always looking the best for our students and faculty.

It seems clear that our School of Medicine will have to take care of many aspects of its community because of the pandemic’s damage: academic issues, mental health, survivor’s condition and mourning for our loss, post-traumatic syndrome, national and global economic problems affecting our country, fear to new waves of the disease, and so on.

After this ferocious pandemic, we will have to take special care of the scars and to embrace the new community we will be.

## Conclusion

The health crisis carries a high degree of uncertainty and implies the need for adaptations and innovations not easily achieved.

In the case of UNAM School of Medicine, the response was swift and forceful, based on the protection of its community, and addressed to continuing with its substantive functions.

The reorganization of goals and processes supported by technological tools allowed us to continue with teaching, research, culture, and leisure activities, although it was different.

Personal relationships between members of the community were also substantially enriched, highlighting the altruistic vocation and the deep sense of solidarity and service in response to the pandemic.

The organization and adaptability of UNAM School of Medicine allowed the implementation of multiple strategies facilitating the resolution of the educational and administrative challenges caused by the pandemic, as well as the creation of new projects for the protection of their academic community and the general Mexican population.

Providing accurate and truthful information about the pandemic to different population groups gave a degree of certainty. We still need to improve, but we have learned that effective communication and respect are crucial to moving forward.

The portrayals for the consequences of this pandemic (political, economic, cultural, personal, bioethical, and social) are enormous. However, it requires innovations and the joining of efforts to overcome the problems that are coming.

Mexico is currently beginning Phase 3 of the pandemic, so we continue to prepare for the difficult days to come. UNAM School of Medicine is ready to continue advancing and reinventing itself during this pandemic to fulfill its mission and vision.

The staggered return to face-to-face work should be planned carefully from now and be fully operative when the health crisis ends.

We believe that when this situation ends, we will use these new lessons and experiences to improve our School of Medicine, transitioning into a more robust, united, and enriched community in the face of the crisis caused by a pandemic.

## Take Home Messages

• Schools of Medicine must adapt to provide educational services during a global health crisis.
• Use of technological tools and effective communication are crucial.
• Effort and social accountability allowed implementation of multiple strategies to face this health crisis.
• Our most challenging situations were effective communication, resistance to migrate from face-to-face to remote activities; technological development; training to implement work and study online; digital gap, computer and internet access; online evaluations; practical skills online evaluation and student withdrawal from clinical clerkships.
• Lessons learned during the pandemic must be used to improve and enrich the academic community in all the possible approaches.

## Notes On Contributors

**Maria de los Angeles Fernandez-Altuna.** General Physician and Ph.D. in Sociomedical Sciences. Currently, Public Health and Basic and Clinical Sciences Integration teacher. Vice Dean for Academic Management Affairs at National Autonomous University of Mexico’s School of Medicine. ORCID ID: https://orcid.org/0000-0002-7990-3856

**Diego Gutierrez Rayon.** General Physician. Public Health and Basic and Clinical Sciences Integration teacher. Head of Integration, Information and Data Analysis Unit at the National Autonomous University of Mexico’s School of Medicine Academic Management Affairs Secretariat. ORCID ID: https://orcid.org/0000-0003-1063-7075

**Mariela Ramirez Resendiz.** General Physician. Head of the Undergraduate Unit at the National Autonomous University of Mexico’s School of Medicine Academic Management Affairs Secretariat. ORCID ID: https://orcid.org/0000-0003-2418-6455

**Patricia Cruz Mendez.** General Physician. Currently works at the Re-engineering and Substantive Processes Improvement Area at the National Autonomous University of Mexico’s School of Medicine Academic Management Affairs Secretariat. ORCID ID: https://orcid.org/0000-0001-6024-4136

**Karla Alejandra Tovar Lopez.** General Physician. Currently contributes to the Re-engineering and Substantive Processes Improvement Area at the National Autonomous University of Mexico’s School of Medicine Academic Management Affairs Secretariat. ORCID ID: https://orcid.org/0000-0002-2212-7884
