# Supplementary material for: Experience of the biggest School of Medicine in Mexico during the COVID-19 pandemic
Source: MedEdPublish (2016). 2020 Nov 6;9:101. Originally published 2020 May 18. [Version 2] doi: 10.15694/mep.2020.000101.2 (PMC10712577; doi:10.15694/mep.2020.000101.2)
Supplement: Peer review history

# Reviewer report: Reviewer response for version 2

**DOI:** 10.21956/mep.20268.r31459

**Published:** 2021-03-20

**Authors:** Megan Anakin

**Affiliations:** 1 University of Otago

This review has been migrated. The reviewer awarded 4 stars out of 5 Thank you for publishing this revised version of your article. I appreciated reading about the authors’ experiences of implementing changes due to the impact of COVID19 on their medical programme. I have read both versions of this article and I agree with the other reviewers that you have adequately addressed their major concerns. Like Ken and Haipeng, I too look forward to learning about how your institution and students have persevered since you provided this description. In particular, I would appreciate an update that further describes Phase 3 and further impacts of COVID19 on the programme. In the lessons learned section, I would appreciate further thoughts about how these lessons might apply to others in different national contexts and how you might plan to evaluate these changes.

Reviewer Expertise:

NA

No decision status is available

# Reviewer report: Reviewer response for version 2

**DOI:** 10.21956/mep.20268.r31460

**Published:** 2021-02-28

**Authors:** Haipeng Xiao

**Affiliations:** 1 The First Affiliated Hospital, Sun Yat-sen University

This review has been migrated. The reviewer awarded 3 stars out of 5 The authors should be congratulated for sharing their important work. Albeit an opinion piece, the Mexican experience should be included in the global "Response to COVID-19" pool of evidence. The manuscript is clear-structured and well-written. Personally, I would like to learn more about the outcomes of these measures in detail. Looking forward to the updates from the authors.

Reviewer Expertise:

NA

No decision status is available

# Reviewer report: Reviewer response for version 2

**DOI:** 10.21956/mep.20268.r31457

**Published:** 2021-02-01

**Authors:** Ken Masters

**Affiliations:** 1 Sultan Qaboos University

This review has been migrated. The reviewer awarded 4 stars out of 5 This paper is Version 2 of an already-strong paper. Although there are opportunities for small areas of improvement, the authors have adequately addressed the major concerns of the previous version’s reviewers, and are thanked for doing so.I look forward to seeing updates on the authors’ work in this area.

Reviewer Expertise:

NA

No decision status is available

# Reviewer report: Reviewer response for version 2

**DOI:** 10.21956/mep.20268.r31458

**Published:** 2020-11-09

**Authors:** Judy McKimm

**Affiliations:** 1 Swansea University

This review has been migrated. The reviewer awarded 3 stars out of 5 Many thanks to the writing team for revising the paper and resubmitting it, I think the inclusion of more emphasis on social accountability improves this. I feel that this is a helpful summary of all the activities carried out at the School in response to the pandemic, and particularly useful for others in low resource settings, although the vast majority of the issues discussed are common to most countries and settings. Where I think this could be improved is as follows:(1) some attention to proof reading and English(2) defining clearly what can be evidenced and what is opinion or assumption - I know this is an opinion piece, however it is helpful to avoid unfounded assumptions if possible (3) perhaps condensing and trimming for succinctness to make it easier to read and followHowever, I think this is interesting and huge congratulations for your perseverance during this difficult time in Mexico, especially in the giving back and supporting local communities.

Reviewer Expertise:

NA

No decision status is available

# Reviewer report: Reviewer response for version 1

**DOI:** 10.21956/mep.19134.r27635

**Published:** 2020-05-19

**Authors:** P Ravi Shankar

**Affiliations:** 1 American International Medical University

This review has been migrated. The reviewer awarded 4 stars out of 5 This is an interesting article about the response of the National Autonomous University of Mexico to the COVID-19 pandemic. The article lists various initiatives which have been carried out both by the university and the medical school. The medical school has put in place a comprehensive set of measures for both its internal and also for the general community. The university seems well-placed technologically to deal with the challenges of the pandemic. The authors provide a long list of measures which have been undertaken. The response of the university will be of special interest to educators and administrators from other universities in developing countries. Some language corrections may be required throughout the manuscript and I can sympathize with the authors whose first language is not English and neither is English, the language of education and communication.

Reviewer Expertise:

NA

No decision status is available

# Reviewer report: Reviewer response for version 1

**DOI:** 10.21956/mep.19134.r27636

**Published:** 2020-05-19

**Authors:** Judy McKimm

**Affiliations:** 1 Swansea University

This review has been migrated. The reviewer awarded 4 stars out of 5 Thank you for writing this article which reflects the experience of a Mexican school of health sciences during the pandemic. I very much like that this is very much contextualised within the societal/community context and reflects the wider obligations of the school. For this reason, maybe some more reference to social accountability might strengthen this aspect. I think this is a good case study of your approach to the pandemic crisis, and you identify lessons learned and key actions taken. It might be useful to put these into a table or figure for ease of reading as there was rather a long set of lists, but the content and suggestions will be useful for others I believe. I think some attention to the key messages and the formatting therefore would enhance this and make the piece stronger. A minor point, there are some slang terms and abbreviations that should be more 'academic' in the writing. And finally, well done on all the actions and positive communications and lessons learned, this will be of use to others facing similar challenges.

Reviewer Expertise:

NA

No decision status is available
